# Association between Objectively Measured Physical Activity and Gait Patterns in People with Parkinson's Disease: Results from a 3-Month Monitoring

**DOI:** 10.1155/2018/7806574

**Published:** 2018-10-17

**Authors:** Micaela Porta, Giuseppina Pilloni, Roberta Pili, Carlo Casula, Mauro Murgia, Giovanni Cossu, Massimiliano Pau

**Affiliations:** ^1^Department of Mechanical, Chemical and Materials Engineering, University of Cagliari, Cagliari, Italy; ^2^A.O.B. “G. Brotzu” General Hospital, Cagliari, Italy; ^3^Department of Life Sciences, University of Trieste, Trieste, Italy

## Abstract

**Background:**

Although physical activity (PA) is known to be beneficial in improving motor symptoms of people with Parkinson's disease (pwPD), little is known about the relationship between gait patterns and features of PA performed during daily life.

**Objective:**

To verify the existence of possible relationships between spatiotemporal and kinematic parameters of gait and amount/intensity of PA, both instrumentally assessed.

**Methods:**

Eighteen individuals affected by PD (10F and 8M, age 68.0 ± 10.8 years, 1.5 ≤ Hoehn and Yahr (H&Y) < 3) were required to wear a triaxial accelerometer 24 h/day for 3 consecutive months. They also underwent a 3D computerized gait analysis at the beginning and end of the PA assessment period. The number of daily steps and PA intensity were calculated on the whole day, and the period from 6:00 to 24:00 was grouped into 3 time slots, using 3 different cut-point sets previously validated in the case of both pwPD and healthy older adults. 3D gait analysis provided spatiotemporal and kinematic parameters of gait, including summary indexes of quality (Gait Profile Score (GPS) and Gait Variable Score (GVS)).

**Results:**

The analysis of hourly trends of PA revealed the existence of two peaks located in the morning (approximately at 10) and in the early evening (between 18 and 19). However, during the morning time slot (06:00–12:00), pwPD performed significantly higher amounts of steps (4313 vs. 3437 in the 12:00–18:00 time slot, *p* < 0.001, and vs. 2889 in the 18:00–24:00 time slot, *p*=0.021) and of moderate-to-vigorous PA (43.2% vs. 36.3% in the 12:00–18:00 time slot, *p*=0.002, and vs. 31.4% in the 18:00–24:00 time slot, *p*=0.049). The correlation analysis shows that several PA intensity parameters are significantly associated with swing-phase duration (rho = −0.675 for sedentary intensity, rho = 0.717 for moderate-to-vigorous intensity, *p* < 0.001), cadence (rho = 0.509 for sedentary intensity, rho = −0.575 for moderate-to-vigorous intensity, *p* < 0.05), and overall gait pattern quality as expressed by GPS (rho = −0.498 to −0.606 for moderate intensity, *p* < 0.05) and GVS of knee flexion-extension (rho = −0.536 for moderate intensity, *p* < 0.05).

**Conclusions:**

Long-term monitoring of PA integrated by the quantitative assessment of spatiotemporal and kinematic parameters of gait may represent a useful tool in supporting a better-targeted prescription of PA and rehabilitative treatments in pwPD.

## 1. Introduction

In people with Parkinson's disease (pwPD), walking dysfunctions represent a very common and disabling feature which is typically expressed by a gait pattern characterized by short stride length, increased cadence, and reduced velocity [1]. Such issues tend to further deteriorate with the progression of the disease [2], thus limiting the ability of the affected individual to perform daily activities and severely reducing the quality of life [3].

Although physical activity (PA) has been found to be beneficial in improving mobility in pwPD [4, 5], they may be reluctant to engage in structured or unstructured PA programs, owing to their increased motor difficulties which tend to favor a sedentary lifestyle. This originates a vicious circle since physical inactivity further negatively affects several clinical domains of PD [6, 7]. Thus, a detailed assessment of both the amount and intensity of PA performed represents a critical issue in evaluating the effectiveness of programs and trials aimed to improve mobility in pwPD. To this end, several studies have attempted to employ objective measurements (e.g., using pedometers or accelerometers) [8] to replace or integrate self-reported data collected using questionnaires [9], which may not adequately reflect the actual activity carried out by pwPD [10]. The availability of continuous quantitative data on mobility has made it possible to precisely identify what aspects of the disease are most involved in PA levels [11, 12], their relationship to history of falls [13], and cognition, depression, and quality of life [14, 15]. However, data collection is usually limited to few days or a week, while long-term monitoring appears to be infrequent, probably owing to compliance issues.

While there is a certain consensus on the fact that PA contributes to improving gait and mobility [5, 16], it is noteworthy that most studies that consider gait parameters as the primary outcome only consider few aspects of them (usually gait speed and cadence) mainly assessed using timed tests. In contrast, few data are available on the whole kinematics of the gait pattern (i.e., spatiotemporal parameters, kinematics, and range of motion during gait of hip, knee, and ankle joints), acquired with state-of-the-art technologies such as motion-capture systems or inertial sensors. Such information would be of interest to better understand the complex pathophysiology of gait disturbance in PD [17] and to assess the effects of neurosurgical, pharmacological, and rehabilitative treatments [18]. Summarizing, the main drawbacks of the study performed to date to investigate the effects of PA on gait in pwPD are the following: the limited period of PA monitoring and the limited number of gait parameters assessed to relate PA to mobility.

To partly overcome such limits, this study aims firstly to describe the patterns of PA in a cohort of pwPD based on a 3-month monitoring. Then, during the same period, quantitative data on the quality of gait patterns, by means of spatiotemporal and kinematic parameters, were also collected and correlated with PA indicators. The hypothesis to verify is that individuals who exhibit better gait features are characterized by higher and more intense PA during their daily lives.

## 2. Methods

### 2.1. Participants

The study was performed in the period March–December 2017 and involved 18 outpatients with PD (10 females and 8 males) followed up at the Neurology Department of the G. Brotzu General Hospital (Cagliari, Italy) who were enrolled on a voluntary basis. Their demographic and clinical characteristics are shown in Table 1.

All participants met the following criteria: diagnosis of PD according to the UK Brain Bank criteria [19]; ability to walk independently; absence of significant cognitive impairment (i.e., Mini-Mental Status Examination (MMSE) > 24; Frontal Assessment Battery (FAB) > 13); absence of psychiatric or severe systemic illnesses; and mild-to-moderate disability assessed by means of the modified Hoehn and Yahr (H&Y) staging scale (1.5 ≤ H&Y < 3). At the time of enrollment, the pharmacologic treatment included levodopa for all participants and MAO-B inhibitors for 11 of them (*n*=8 had rasigiline and *n*=3 had safinamide). The study was carried out in compliance with the ethical principles for research involving human subjects expressed in the Declaration of Helsinki and was approved by the local ethics committee (Prot. PG/2014/19654). All participants signed an informed consent form after a detailed explanation of the purposes of the study and the methodology used in the experimental tests.

### 2.2. Data Collection and Processing: Physical Activity

Data on PA were collected using a triaxial accelerometer (ActiGraph GT3X; Acticorp Co., Pensacola, FL, USA) previously employed in similar studies carried out on individuals with PD [20–22]. During the first meeting with participants, their anthropometric data required to initialize the device (i.e., stature and body mass) were recorded using an ultrasonic digital height meter (Soehnle 5003; Soehnle, Germany) and a digital scale (RE310; Wunder, Italy). Each participant was then asked to wear the accelerometer on the nondominant wrist for 3 months 24 h/day and instructed to remove it only for showering, bathing, and any other water-based activities (i.e., swimming). The choice of the wrist as the site of placement was made to increase wear time compliance and provide data on sleep [23–25]. The devices were set to collect data using 60 s epochs and 30 Hz frequency and were usually operative for at least 30 days before the battery ran out of charge. At that point, the participants came back to the laboratory to download the acquired data and charge the accelerometer. At the end of the third month, raw data were processed using ActiLife® software v6.13.3 to perform step counts and PA classification based on the cut-points defined by Hildebrand et al. [26], Wallén et al. [20], and Nero et al. [21] for the acceleration vector magnitude (VM) defined as follows:

(1) $$\begin{array}{c} \text{VM}=\sqrt{x^{2}+y^{2}+z^{2}}, \end{array}$$

where *x*, *y*, and *z* are the accelerations recorded by the device in each of the three directions.

The use of 3 different processing procedures, although all based on the same device and the same physical variable (i.e., VM), was suggested by the fact that, to date, a validated set of cut-points for wrist placement of the accelerometer in individuals with PD is unavailable. Thus, the algorithm of Hildebrand et al. [26] was chosen because it is the only one available for wrist-worn data acquisition on elderly people, while the algorithms of Wallén et al. [20] and Nero et al. [21] were previously validated for individuals with PD but in the case of hip placement. In particular, the Nero algorithm provides a set of cut-points for different walking speeds. All the PA parameters were then grouped by considering the following three time slots, namely, 6:00–12:00 (TS 1, morning), 12:00–18:00 (TS 2, afternoon), and 18:00–24:00 (TS 3, evening). The acquired data were considered valid if wear time amounted to at least 16 h/day, by considering nonwear time as a time interval of at least 60 consecutive minutes of zero accelerometric counts.

### 2.3. Data Collection and Processing: 3D Gait Analysis

A 3D computerized gait analysis was performed at the beginning (T0) and at the end (T3) of the 3-month evaluation period to calculate both spatiotemporal and kinematic gait parameters using an optoelectronic system composed of 8 infrared cameras (Smart-D; BTS Bioengineering, Italy) set at a frequency of 120 Hz. After anthropometric data collection, 22 spherical retroreflective passive markers (14 mm in diameter) were placed on the skin of the individual's lower limbs and trunk at specific landmarks, following the protocol described by Davis et al. [27]. Participants were then asked to walk barefoot at a self-selected comfortable speed in the most natural manner possible on a 10 m walkway for at least six times, allowing suitable rest times between the trials. The raw data were then processed with the Smart Analyzer (BTS Bioengineering, Italy) dedicated software to calculate the following:

- Five spatiotemporal parameters (gait speed, cadence, stride length, stance, and swing-phase duration)
- Nine kinematic parameters, namely, pelvic tilt, rotation, and obliquity, hip flexion-extension, adduction-abduction, and rotation, knee flexion-extension, ankle dorsi-plantarflexion, and foot progression (i.e., the angle between the axis of the foot and the walking direction)
- Dynamic range of motion (ROM) for hip and knee flexion-extension and ankle dorsi-plantarflexion calculated during the whole gait cycle as the difference between the maximum and minimum values of each angle recorded during a trial

Kinematic data were summarized using the Gait Variable Score (GVS) and the Gait Profile Score (GPS), which are concise measures of gait quality proposed by Baker et al. [28]. Although originally proposed for children with cerebral palsy, this approach was found to be effective in characterizing gait alterations in individuals with PD [29, 30]. Specifically, the GVS represents the root mean square (RMS) difference between the tested subject's curve for a certain movement of the nine previously listed parameters (e.g., knee flexion-extension) and a reference curve calculated as the mean value of tests performed on the unaffected subjects. The GPS combines the nine GVS values in a single score, which indicates the degree of deviation from a hypothetical “normal” gait (i.e., the larger the GPS, the less physiological the gait pattern); values for healthy individuals lie in the range 5-6° [31]. In the present study, the reference data were obtained from a database of healthy individuals of the same age range of the subjects tested here, available from the Smart Analyzer software.

### 2.4. Statistical Analyses

The possible differences in PA levels associated with each time slot were assessed using the one-way analysis of variance for repeated measures (RM-ANOVA) considering as the independent variable the time slot and as dependent variables the PA parameters. The level of significance was set at *p* < 0.05, and effect sizes were assessed using the eta-squared coefficient (*η*^2^). Influence of time on spatiotemporal and kinematic parameters of gait was assessed using one-way RM-ANOVA considering time (T0, T3) as the independent variable and the previously listed gait parameters as dependent variables. All analyses were performed using the IBM SPSS Statistics v.20 software (IBM, Armonk, NY, USA). Finally, the relationship between PA and gait parameters was explored using Spearman's rank correlation coefficient, again by setting the level of significance at *p* < 0.05.

## 3. Results

**Figure 1:**
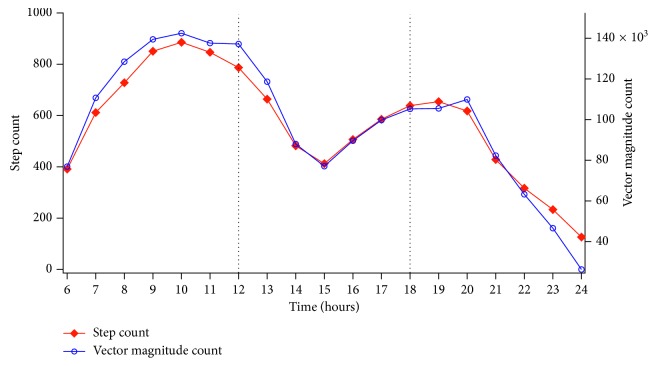
Hourly trend (average value of 3 months) of step counts and vector magnitude counts.

**Figure 2:**
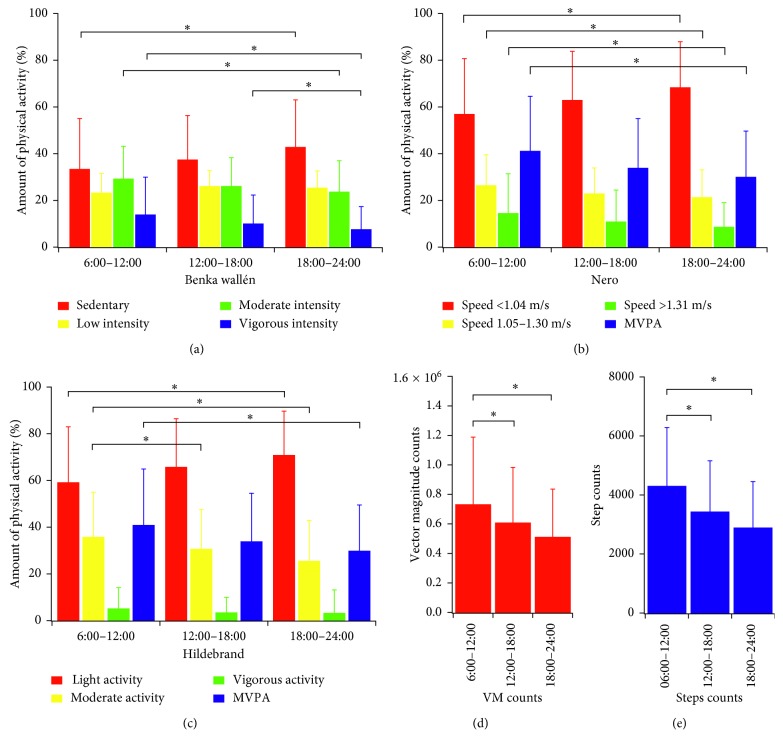
Physical activity amount classified as a function of intensity for the 3 time slots. (MVPA = moderate‐to‐vigorous physical activity; VM = vector magnitude).

The hourly trends of step count and VM and the mean values of PA classified as a function of its intensity calculated on the 3 selected time slots are illustrated in Figures 1 and 2, while Table 2 shows the classification of PA parameters according to the cut-points defined by the 3 algorithms previously described.

ANOVA revealed a significant main effect of the time slot for both step counts (*F*(2,17) = 9.81, *p* < 0.001, *η*^2^ = 0.11) and VM counts (*F*(2,17) = 9.76, *p* < 0.001, *η*^2^ = 0.01). The highest values for both variables were observed in the 6:00–12:00 time slot, while participants appeared to be less active in the evening.

The results of the classification of PA intensity with the three algorithms employed show similar results. For the Hildebrand algorithm, the effect of time slots was significant for percentage of time spent in sedentary activity (*F*(2,17) = 8.22, *p* < 0.001, *η*^2^ = 0.07), low intensity (*F*(2,17) = 4.73, *p*=0.015, *η*^2^ = 0.05), and moderate-to-vigorous intensity (MVPA) (*F*(2,17) = 6.57, *p*=0.004, *η*^2^ = 0.06), with sedentary behavior being reduced in the morning and increased in the evening (59.1% vs. 70.9%, *p* < 0.001) and, conversely, higher MVPA in the morning (40.9% vs. 29.9%, *p*=0.003). The Wallén algorithm yielded a significant effect of time for all the intensity levels: sedentary (*F*(2,17) = 4.67, *p*=0.017, *η*^2^ = 0.05), low intensity (*F*(2,17) = 3.43, *p*=0.044, *η*^2^ = 0.05), moderate intensity (*F*(2,17) = 4.16, *p*=0.024, *η*^2^ = 0.05), and MVPA (*F*(2,17) = 7.42, *p*=0.002, *η*^2^ = 0.05). Finally, for the Nero algorithm, which classifies PA intensity in terms of gait speed, time was found to significantly influence the percentage of time spent at speeds below 1.04 m/s (*F*(2,17) = 7.55, *p*=0.002, *η*^2^ = 0.06), at speeds in the range 1.05–1.30 m/s (*F*(2,17) = 3.79, *p*=0.033, *η*^2^ = 0.05), and at speeds above 1.31 m/s (*F*(2,17) = 5.36, *p*=0.009, *η*^2^ = 0.04). Even in this case, the morning represents the time of day characterized by higher percentages of time spent at the higher gait speed (14.6% vs. 8.7% in the evening, *p*=0.008).

Spatiotemporal and kinematic parameters of gait did not vary significantly between the beginning and the end of the 3-month period, except for the GVS of pelvic tilt, as visible from data in Tables 3 and 4.

To conclude, Tables 5 and 6 show the results of the correlations between PA and gait variables.

Duration of the swing phase and cadence were found to be the gait variables significantly correlated with a larger number of PA parameters regardless of the algorithm considered (11 to 14 significant correlations out of 15 possible). Stride length was found to be significantly correlated only with step counts (rho = 0.59) and percentage of sedentary activity (rho = −0.48) calculated according to Wallén, while no significant correlations were found for gait speed. As regards the kinematic variables, the GPS was found to be significantly correlated negatively with the percentage of moderate activity as calculated by the Wallén (rho = −0.61) and Hildebrand (rho = −0.50) algorithms. The GVS associated with knee flexion-extension was also found to be negatively correlated with the percentage of moderate activity according to Wallén (rho = −0.54) and with the percentage of time spent at walking speed between 1.05 and 1.30 m/s (Nero algorithm, rho = −0.53). Dynamic ROM of the knee was negatively correlated with sedentary activity (Wallén algorithm, rho = −0.47) and positively correlated with vigorous activity (Hildebrand algorithm, rho = 0.49). Finally, step count was found to be positively correlated with both dynamic ROMs of the hip and knee (rho = 0.50 and 0.57, respectively).

## 4. Discussion

### 4.1. Hourly Trends of PA

The aim of the present study was to perform long-term monitoring of PA in pwPD and investigate the existence of possible correlations between PA and gait parameters, with these being objectively assessed using the gold standard for quantitative analysis of human motion, namely, the motion capture system. Our results detected a pattern for PA of pwPD with low-mild disability. They clearly show the existence of two peaks of PA, one in the morning (approximately hour 10) and another in the evening located between 6 and 7 PM. Unfortunately, a direct comparison with previous studies is difficult because even though several of these continuously monitored PA, they mostly report only examples of the curves of variation of PA parameters (usually the number of steps) during the day [31, 32]. To our knowledge, only the recent study by Cai et al. [12] calculated a mean curve of variation for step counts calculated for a sample of 21 pwPD, but their data appear not to suggest the existence of a well-defined pattern. However, our results do appear to be partly consistent with those reported in two studies performed on healthy older adults [33, 34], both of which observed a marked peak in PA (expressed in terms of either steps or accelerometric counts) approximately at 10. Sartini et al. [33] detected a second peak located approximately at 14-15, while in the study by Valenti et al. [34], activity appears to monotonically decrease from 10 until night. As previously mentioned, in the present study, a second relevant PA peak was found between 18 and 19 and thus later in the day with respect to Sartini et al. [33]. Such differences can probably be explained by the fact that, in our case, PA was mostly monitored during months characterized by favorable environmental conditions (Cagliari has a mild climate for most of the year) which probably encouraged participants to walk or spend time outdoor in the evening as well. In any case, it must be noted that when PA is analyzed on the basis of the 3 defined time slots, our results appear to be fully consistent also with those of Valenti et al. [34] who also detected a significant reduction in PA in the evening time slots.

### 4.2. Comparison between Different Algorithms for PA Intensity Classification

**Figure 3:**
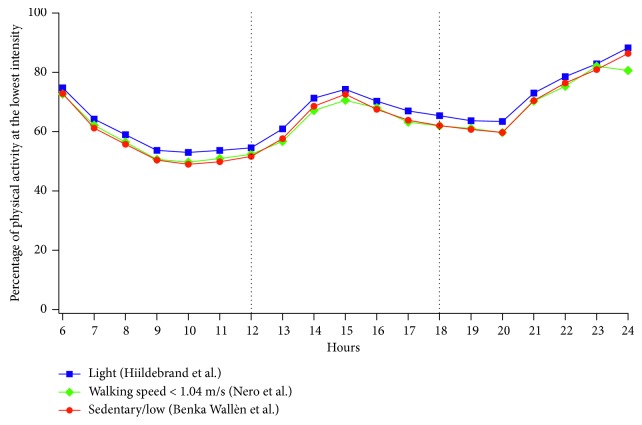
Hourly trends for lowest-intensity physical activity (<3 MET) as calculated using 3 different cut-point sets.

One of the purposes of our study was to compare different algorithms previously validated for use in pwPD [20, 21] but designed for waist placement of the accelerometer and one calibrated on older adults [26] in the case of nondominant wrist placement. The results show that, despite the different cut-points and the correction applied by the ActiLife® software for wrist placement with algorithms designed for waist placement, the hourly trend for the different intensities appears to be very similar. For example, Figure 3 shows a comparison between the hourly trends for the lowest PA intensity (i.e., <3 MET), namely, sedentary/light intensity [20], walking speed <1.04 m/s [21], and light intensity [26], which demonstrates the good agreement of the 3 algorithms.

### 4.3. Correlation between PA and Gait Parameters

The most innovative aspect of the present study is represented by the search for possible correlations between PA and gait patterns, with the latter being investigated using 3D computerized gait analysis, which represents the gold standard for human movement analysis. The results show that cadence and swing-phase duration exhibit the highest number of significant correlations with amount and intensity of PA performed. Individuals who spent less time in sedentary behavior and more time in moderate-to-vigorous activity are likely to exhibit a gait pattern characterized by reduced cadence and increased swing phase. Instead, the relationship of PA intensity with both stride length and stance-phase duration appears to be less generalized.

The reduction in swing-phase duration, which is a physiologic sign of gait deterioration associated with aging [35] and further worsened in pwPD [30], is a cofactor involved in the risk of falls [36]. However, it has been demonstrated that exercise (for healthy older adults [37]) or specific gait training integrated with rhythmic auditory stimulation (for pwPD [38]) can partly reverse this negative trend. In this context, our data suggest that pwPD engaged in higher and more intense levels of PA are characterized by increased swing-phase duration and thus, indirectly, probably exposed to a lower risk of falls.

Cadence has been recognized as one of the gait parameters most suitable for representing ambulatory activity in free living, and in young healthy individuals, it has been found to be strongly correlated with PA intensity [39]. Our results suggest that, in our cohort of pwPD, participants with higher baseline cadence tend to spend more time in sedentary/low-intensity behavior, while those characterized by lower baseline cadence are more likely to engage in moderate-to-vigorous activity. Although there is no specific evidence about the role of cadence in preventing/promoting PA engagement, it is possible to hypothesize that individuals with higher (e.g., above normality) cadence are also those who experience increased difficulties in optimally managing the stride length-cadence relationship [40] and thus are more likely to exhibit a disturbed gait which somehow discourages them from being active. This may also partly explain why we also detected more marked sedentary behavior (at least with the Wallén approach) in pwPD with shorter steps. In brief, individuals who walk with shorter steps/higher cadence appear to be characterized by prevalent sedentary/low-intensity PA.

Finally, the overall quality of the gait pattern, as expressed by GPS, appears to be moderately correlated with the percentage of time spent in moderate-intensity PA, consistent in all the tested approaches; in particular, the alterations at the knee joint level appear to be the most involved in this process. Previous studies highlighted the existence of alterations of knee flexion-extension during gait, especially in terms of inadequate extension in the stance phase [30]. This is probably due to reduced muscle strength in the knee extensors, a phenomenon commonly observed in pwPD [41, 42], which can also be the result of impairment of dynamic stability [43]. Such results suggest that a detailed analysis of the role of walking abilities in PA features cannot be based solely on the study of spatiotemporal parameters but should also take into account possible kinematic alterations.

Some limitations of the study are to be acknowledged. Firstly, the participants were all volunteers, as the particular nature of the study (i.e., long-term use of a wearable device 24 h/day) required high levels of compliance to achieve reliable results [44]. Secondly, the tested sample was composed of a very homogeneous group of highly motivated individuals (as shown by their good PA performance) with low-mild disability living in an inner city residential area. Such biases make it difficult to generalize our results [45] particularly to different geographic and socioeconomic contexts and to individuals with PD more severely impaired. Also, it should be considered that the gait parameters were acquired in the laboratory with participants undressed and barefoot, while PA was assessed under daily living conditions; thus, the measurement conditions are obviously quite different.

## 5. Conclusion

This study investigated the relationship between amount and intensity of PA performed by individuals affected by PD with low-mild disability (objectively assessed using wrist-worn triaxial accelerometers) and the kinematic features of their gait patterns provided by computerized 3D gait analysis. PA parameters were estimated using different sets of cut-points for the accelerometric counts previously validated on pwPD and healthy older adults. The results show a daily trend, described similarly by all the approaches tested, characterized by two distinct peaks of activity, located in the morning and early evening. The main hypothesis of the study, namely, the existence of a relationship between the quality of the gait pattern and amount/intensity of performed PA, was substantially confirmed by the results of the correlation analysis. In particular, higher and more intense activity appears to be related to swing-phase duration and cadence, while the percentage of time spent in moderate activity also appears to be associated with the overall quality of gait kinematics (expressed by means of the GPS summary index) and with the alteration of flexion-extension of the knee joint. Although further studies on larger cohorts are necessary to better elucidate the influence of the disability level, gender, and socioeconomic status, the findings of the present study suggest that the continuous monitoring of PA in pwPD may represent a useful tool in predicting possible changes in the gait pattern and verify the effectiveness of rehabilitative treatments and PA programs.

## Figures and Tables

**Table 1:** Anthropometric and demographic aspects of participants.

Variable		Mean ± SD	Range (min–max)
Age (years)		68.0 ± 10.8	53–83
Height (cm)		165.6 ± 7.9	150–178
Body mass (kg)		69.2 ± 9.4	50–81
PD duration (years)		9.9 ± 6.0	4–27
Hoehn and Yahr (H&Y)		1.9 ± 0.4	1.5–2.5
Unified Parkinsonʼs Disease Rating Scale (UPDRS III)	Overall score	17.8 ± 9.6	5–32
	Axial subscore (items 27–30)	2.8 ± 1.5	1–5

Values are expressed as mean ± SD.

**Table 2:** Physical activity patterns for the morning, afternoon, and evening time slots calculated as means of the 3-month monitoring period.

Physical activity patterns				
		TS 1 (hours 6–12)	TS 2 (hours 12–18)	TS 3 (hours 18–24)
Wallén et al. [20]	Sedentary behavior (%)	33.41 ± 21.66	37.62 ± 18.70	42.78 ± 21.28^a^
	Low intensity (%)	23.09 ± 8.55	26.01 ± 6.72	25.59 ± 6.91
	Moderate intensity (%)	29.32 ± 13.85	26.21 ± 12.03	23.79 ± 13.05^a^
	Vigorous intensity (%)	13.93 ± 16.06	10.08 ± 12.21^a^	7.66 ± 9.70^a^
	MVPA^*∗*^ (%)	43.25 ± 20.31	36.29 ± 18.50^a^	31.45 ± 16.53^a^

Nero et al. [21]	Speed ≤ 1.04 m/s (%)	57.08 ± 26.65	62.99 ± 20.81	68.39 ± 19.62^a^
	Speed 1.05–1.30 m/s (%)	26.53 ± 13.00	23.00 ± 10.86	21.38 ± 11.60^a^
	Speed ≥ 1.31 m/s (%)	14.65 ± 16.82	10.96 ±13.42	8.68 ± 10.39^a^
	MVPA (%)	41.18 ± 23.47	33.96 ± 21.10	30.07 ± 19.56^a^

Hildebrand et al. [26]	Light intensity (%)	59.05 ± 23.93	65.97 ± 20.41^a^	70.87 ± 18.75^a^
	Moderate intensity (%)	35.77 ± 19.10	30.58 ± 16.87	25.54 ±17.25^a^
	Vigorous intensity (%)	5.18 ± 8.91	3.44 ± 6.54	3.22 ± 9.86
	MVPA^*∗∗*^ (%)	40.95 ± 23.93	34.03 ± 20.41	29.91 ± 19.59^a^
	Steps counts (daily steps)	4313 ± 1973	3437 ± 1719^a^	2889 ± 1557^a^
	Vector magnitude (counts per day)	735639 ± 452680	610262 ± 372729^a^	512835 ± 323037^a,b^

Values are expressed as mean ± SD. MVPA: moderate-to-vigorous physical activity; TS: time slot; ^a^significant difference vs. TS 1; ^b^significant difference vs. TS 2; ^*∗*^sum of moderate and vigorous intensity; ^*∗∗*^sum of light and moderate intensity.

**Table 3:** Values of the spatiotemporal parameters of gait at the beginning and end of the 3-month observation period.

Spatiotemporal parameters of gait			
	T0	T3	*p* value
Step length (m)	0.59 ± 0.10	0.59 ± 0.01	0.824
Gait speed (m/s)	1.18 ± 0.23	1.18 ± 0.19	0.980
Cadence (steps/min)	120.39 ± 11.18	120.88 ± 9.09	0.819
Stance-phase duration (s)	0.60 ± 0.07	0.60 ± 0.05	0.754
Swing-phase duration (s)	0.40 ± 0.04	0.39 ± 0.03	0.522

Values are expressed as mean ± SD.

**Table 4:** Values of the kinematic parameters of gait at the beginning and end of the 3-month observation period.

Kinematic parameters of gait				
		T0	T3	*p* value
	GPS (°)	7.31 ± 1.61	7.84 ± 2.44	0.637
GVS (°)	Pelvic tilt	5.51 ± 3.90	7.64 ± 5.10	0.042
	Pelvic rotation	3.80 ± 1.30	4.29 ± 1.79	0.285
	Pelvic obliquity	2.47 ± 1.18	2.67 ± 1.13	0.481
	Hip flexion-extension	8.30 ± 4.09	10.41 ± 6.22	0.059
	Hip abduction-adduction	3.96 ± 1.59	4.15 ± 1.50	0.564
	Hip rotation	8.59 ± 2.70	8.14 ± 2.99	0.641
	Knee flexion-extension	9.17 ± 3.64	9.37 ± 4.13	0.157
	Ankle dorsi-plantarflexion	7.17 ± 2.48	6.22 ± 2.28	0.319
	Foot progression	7.80 ± 3.77	7.89 ± 4.14	0.828

ROM (°)	Hip flexion-extension	42.15 ± 7.45	43.27 ± 7.23	0.197
	Knee flexion-extension	57.39 ± 4.23	57.72 ± 5.28	0.479
	Ankle dorsi-plantarflexion	25.22 ± 6.70	26.47 ± 6.82	0.129

Values are expressed as mean ± SD.

**Table 5:** Spearman's correlation analysis between physical activity intensity and spatiotemporal parameters of gait.

Correlation between physical activity and spatiotemporal parameters of gait						
		Gait speed	Stride length	Cadence	Stance phase	Swing phase
Wallén et al. [20]	Sedentary behavior (%)	−0.088	−0.482^*∗*^	0.509^*∗*^	−0.430	−0.675^*∗∗*^
	Low intensity (%)	0.060	−0.049	0.309	−0.153	−0.361
	Moderate intensity (%)	−0.105	0.159	−0.451	0.374	0.612^*∗∗*^
	Vigorous intensity (%)	0.067	0.423	−0.531^*∗*^	0.427	0.674^*∗∗*^
	MVPA^*∗*^	0.007	0.378	−0.575^*∗*^	0.457	0.717^*∗∗*^

Nero et al. [21]	Speed ≤ 1.04 m/s (%)	−0.009	−0.356	0.591^*∗∗*^	−0.503^*∗*^	−0.687^*∗∗*^
	Speed 1.05–1.30 m/s (%)	−0.104	0.169	−0.534^*∗*^	0.444	0.683^*∗∗*^
	Speed ≥ 1.31 m/s (%)	0.024	0.367	−0.544^*∗*^	0.412	0.669^*∗∗*^
	MVPA (%)	−0.025	0.325	−0.591^*∗∗*^	0.495^*∗*^	0.690^*∗∗*^

Hildebrand et al. [26]	Light intensity (%)	0.007	−0.358	0.575^*∗*^	−0.467	−0.704^*∗∗*^
	Moderate intensity (%)	−0.072	0.291	−0.575^*∗*^	0.474	0.734^*∗∗*^
	Vigorous intensity (%)	0.206	0.514^*∗*^	−0.437	0.313	0.604^*∗∗*^
	MVPA (%)	0.007	0.378	−0.575^*∗*^	0.456	0.717^*∗∗*^
	Step count	0.343	0.586^*∗*^	−0.375	0.239	0.588^*∗*^
	Vector magnitude count	−0.001	0.360	−0.577^*∗*^	0.469^*∗*^	0.704^*∗∗*^

^*∗*^ *p* < 0.05; ^*∗∗*^*p* < 0.001.

**Table 6:** Correlation analysis between physical activity intensity and kinematic parameters of gait.

Correlation between physical activity and kinematic parameters of gait								
		GPS	GVS hip FE	GVS knee FE	GVS ankle DP	ROM hip	ROM knee	ROM ankle
Wallén et al. [20]	Sedentary behavior (%)	0.310	0.019	0.203	−0.106	−0.346	−0.474^*∗*^	−0.424
	Low intensity (%)	0.123	0.239	−0.181	−0.465	−0.038	−0.007	−0.267
	Moderate intensity (%)	−0.606^*∗∗*^	−0.380	−0.536^*∗*^	0.63	0.143	0.276	0.246
	Vigorous intensity (%)	−0.334	−0.099	−0.168	0.205	0.315	0.397	0.329
	MVPA^*∗*^	−0.336	−0.164	−0.135	0.276	0.286	0.373	0.341

Nero et al. [21]	Speed ≤ 1.04 m/s (%)	0.326	0.216	0.143	−0.244	−0.315	−0.381	−0.307
	Speed 1.05–1.30 m/s (%)	−0.576^*∗*^	−0.394	−0.527^*∗*^	0.018	0.195	0.377	0.212
	Speed ≥ 1.31 m/s (%)	−0.275	−0.107	−0.100	0.265	0.282	0.383	0.304
	MVPA (%)	−0.306	−0.217	−0.115	0.272	−0.275	0.370	0.298

Hildebrand et al. [26]	Light intensity (%)	0.327	0.182	0.143	−0.265	−0.276	−0.356	−0.328
	Moderate intensity (%)	−0.498^*∗*^	−0.279	−0.307	0.216	0.207	0.313	0.266
	Vigorous intensity (%)	−0.308	−0.094	−0.156	0.112	0.424	0.490^*∗*^	0.402
	MVPA (%)	−0.336	−0.164	−0.135	0.276	0.286	0.373	0.341
	Step count	−0.184	−0.106	−0.001	0.108	0.503^*∗*^	0.575^*∗*^	0.336
	Vector magnitude count	−0.323	−0.170	−0.150	0.261	0.282	0.362	0.320

^*∗*^ *p* < 0.05; ^*∗∗*^*p* < 0.001.
